# Psychological Problems Related to Infertility

**DOI:** 10.7759/cureus.30320

**Published:** 2022-10-15

**Authors:** Aanchal Sharma, Deepti Shrivastava

**Affiliations:** 1 Department of Obstetrics and Gynaecology, Jawaharlal Nehru Medical College, Datta Meghe Institute of Medical Sciences, Wardha, IND

**Keywords:** psychotherapy, depression, anxiety, art, ivf, infertility

## Abstract

Infertility is a problem of great significance among millions of couples. In our society, to have a child means living a fulfilled life. Thus, couples who cannot do so feel barren and incomplete. Therefore, infertility is more than just a medical problem. It affects all aspects of life, the most important being mental health. For many years, a person's mental health has been overlooked, but in the past few years, it has gained importance that mental health is as significant as physical health, which has also been stated in the WHO definition of health. This article discusses the mental toll that infertility takes on a person's life. A person may experience a myriad of psychological problems, of which stress for extended periods is one. Furthermore, it may cause feelings of guilt, emptiness, anxiety, and depression. The article mainly focuses on the need for counseling for couples dealing with infertility and under treatment because of the long waiting periods accompanying it. Although both men and women can contribute to infertility in India, women are exclusively held responsible, leading to more significant stress while undergoing treatment. The paper also provides an overview of infertility, the factors responsible, and the treatments currently available. Various studies have shown that delivering psychological support through psychiatric clinical specialists may accentuate the results of IVF (In-vitro fertilization) and ART (Assisted Reproductive Technology). This review sheds light on the effects of infertility on quality of life and how it can be prevented or reduced through psychotherapy.

## Introduction and background

In simple language, infertility means the inability to procreate. Infertility is a disease of the male or female reproductive system defined by the failure to achieve a pregnancy after 12 months or more of regular unprotected sexual intercourse [1]. Infertility affects millions of people of reproductive age worldwide - and has an impact on their families and communities. Estimates suggest that between 48 million couples and 186 million individuals live with infertility globally [1]. Most couples have an identifiable cause, and the remaining have unexplained infertility. Female partners contribute to 40%-55% of infertility cases, and male partners contribute to about 20%-40% [1]. Infertility can be either primary or secondary. If there is no history of pregnancy in the past, it is termed primary. If a history of pregnancy in the past is present (irrespective of the outcome of pregnancy), it is called secondary. There are different factors involved that can cause female and male infertility. Female infertility can be due to ovarian cause (most common), tubal cause, uterine cause, cervical cause, or unexplained. One of the ovarian causes is polycystic ovarian syndrome (PCOS), the incidence of which has considerably increased in the past few years. Male infertility may be due to abnormal sperm production, problems in the ejaculation of semen, defects in the motility of sperm, or absence/low levels of sperm. With medical advancements, various infertility treatments now available, and their use has tremendously increased in recent years [1]. This increase can be attributed to delayed childbearing age among women and a rise in success rates. Infertility treatment can be categorized into three groups, i.e., pharmacological, surgical, and Assisted Reproductive Techniques which include in-vitro fertilization (IVF), intrauterine insemination (IUI), assisted hatching, gamete intrafallopian transfer (GIFT) and surrogacy. In vitro fertilization (IVF), which is the fertilization of an ovum in a laboratory environment and implantation of the zygote in a woman’s uterus, as well as intracytoplasmic sperm insertion (ICSI) which precedes the implantation of the embryo in the woman’s body [2]. In conjunction with IVF, Intracytoplasmic Sperm Injection (ICSI) is done by directly injecting a single sperm into each egg retrieved from a woman to attain fertilization. Despite the increasing prevalence of Assisted Reproduction Technologies (ART), there are no uniform standards for psychological and psychiatric procedures in the case of mental health complications of the diagnosis and treatment of infertility [3]. It can be understood that people undergoing infertility treatment must be under a lot of stress. There is a complicated correlation between infertility and psychological stress. On the one hand, a study indicated that infertile couples are subject to greater stress and have an increased risk of developing psychological disorders compared with healthy couples [4]. On the other hand, high levels of psychological distress have been revealed to increase infertility [5].

## Review

Psychological stress due to the diagnosis of infertility

**Figure 1:**
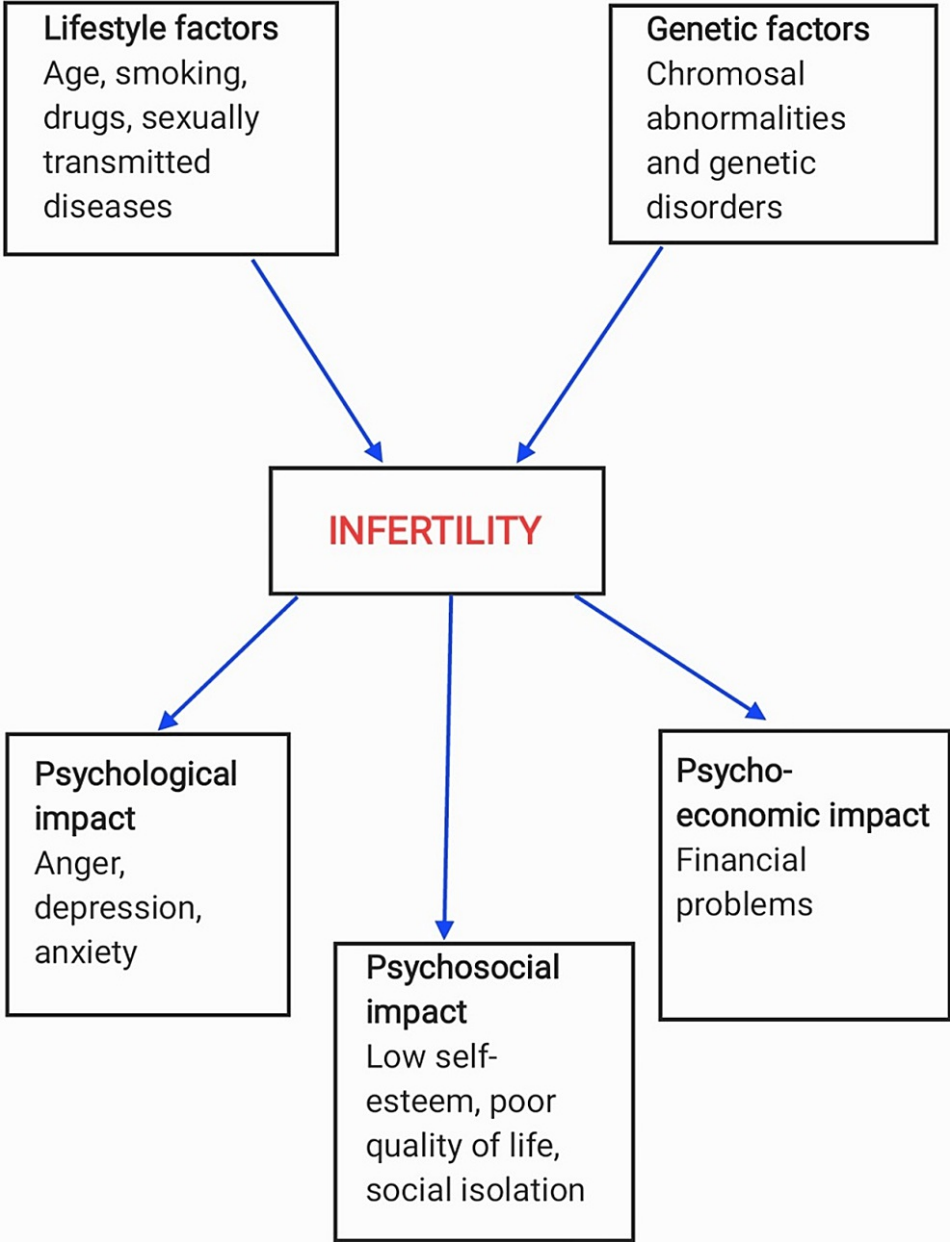
Various ways in which infertility can affect couples

Even though infertility is not a mortal condition, being diagnosed as infertile can be a nerve-racking experience for couples. Infertility can cause psychological distress, emotional stress and financial difficulties for both partners [6]. Couples may feel emotions like anger, guilt, sadness, depression, anxiety, and loss of self-confidence and self-esteem. Apart from this, the financial cost of infertility treatment also significantly contributes to the stress. The average cost for one cycle of IVF in India ranges from INR 1,00,000 to 3,50,000, with the additional cost of medications and tests. Because of such a high cost, some couples cannot get treatment leading to hopelessness (Figure 1). Childbearing is considered necessary in the Indian society, so this social pressure may add to the existing stress. Furthermore, it may also harm a person's relationship with their partner as well as with that of friends and family members. This may result in detachment from the family and decreased social interaction. Psychological stress is also perceived as a potential clinical risk factor that may reduce male fertility [7-9]. Some authors have succeeded in demonstrating that an inverse relationship exists between psychological stress and semen parameters [7-11]. Stress may also have a detrimental effect on the treatment outcome. Figure 1 showing various ways in which infertility can affect couples.

Stress-related to infertility treatment

Couples frequently talk about losing a satisfying sexual life once infertility treatments begin. “Sex on demand”, which is scheduled according to cycles during the month shifts from what was perhaps a spontaneous event to one that is viewed as a task to accomplish [12]. The most prudent decision the couple needs to make is whether they should divulge telling their friends and family about the IVF treatment. The experience of being hopeful only to have hopes dashed by menstruation, perhaps month after month, and the ongoing stress of the variety of invasive physical procedures become common occurrences for the couple being treated for infertility [12]. Besides the mental effects that may arise from infertility, some other side effects may also be caused by drugs and hormones used to treat infertility. For example, the synthetic estrogen clomiphene citrate (Clomid, Serophene), which is often prescribed as it improves ovulation and increases sperm production, can trigger anxiety, interruptions of sleep, and irritability in women [11]. Another drug named, Letrozole (estrogen receptor blocker) can cause dizziness, fatigue, headaches, or breast pain. Lupron may cause acne, anxiety, depression, or mood changes (Table 1). Therefore, it may be difficult for patients and clinicians to understand which responses are psychological and which are caused by medication [13].

Table 1 shows the most commonly used fertility drugs, their use, and common side effects.

**Table 1:** Most commonly used fertility drugs, their use and common side effects [13-15]

Fertility drug	Use	Possible side effects
Clomiphene citrate	It blocks the receptors that are stimulated by estrogen and thus, increases the circulating estrogen.	Mood changes, headache, weight gain, nausea
Letrozole	It works in the same way as clomiphene citrate. In women with Clomid resistance, Letrozole is used.	Fatigue, dizziness, abnormal menstrual bleeding, sleep disturbances
Lupron	It is a GnRH agonist which acts by inactivating the body's natural reproductive system so that the clinician has complete control over ovarian maturation.	Mood swings, anxiety, depression, acne
Oral contraceptives	Used in IVF treatment.	Weight gain, depression

It is noteworthy that the in vitro procedure is not only connected with the uncertainty a couple has about the conception but also with an increased inference in the bodies of the treated people (everyday injections, blood testing, ultrasonography, providing sperm samples, etc.) [2]. 

Stress-related to failed infertility treatment

Infertility procedures help only ~50% of patients to become parents, with this probability of achieving pregnancy lowering with age [16]. This implies that couples who fail in the first round of IVF may need to opt for a second round which not only creates a barrier for financially challenged couples but also takes a swing on a person's mental health. It can be substantially more difficult for women who have endured several miscarriages in the past. The degree of stress is undoubtedly increased, during the entire therapy procedure, by the lack of emotional and educational support. 

Therapies that may help

*Infertility Counseling* 

To assist people in coping with the psychosocial effects of infertility, a qualified medical health practitioner (MHP) offers fertility counseling to those contemplating or receiving reproductive treatments. Counseling can be provided for a couple, individually, or in a group. It aims to address the extraordinary situation-specific needs of patients (such as in times of high distress, in pregnancy after infertility, in multiple pregnancies, while facing the end of medical treatment, while entering third-party donor programs) and is implemented in several formats such as individual, couple, and group [17]. International guidelines [18] propagate that infertility counseling is believed to be different from the usual disease-orientated gynecology and obstetrics consultations as it focuses on the emotional crisis associated with an unfulfilled wish or life goal; the medical treatments required to meet this wish commonly consists of repeated cycles of interventions which have a narrow success rate; the long-lasting wait creates frustration, disappointments, desperation and additional marital, familial, and interpersonal stresses, family and the intracouple dynamics often gets affected as the evaluation and diagnostic procedures impact the intimate lives and personal well-being of couples [18]. 

Role of Psychotherapy

A crucial point for the therapists to contemplate is that couples going through infertility treatment will experience the process individually and as a whole. The couple may not discuss these feelings with each other. Generally, men experience great difficulty expressing their emotions in our society. Because of the individual nature of passing through stages of denial, anger, blame, shame, and despair, many practical problems arise [12]. For couples who do not wish to share their problem with others, a therapist can provide a safe space for them to express emotions. Psychotherapy is an important intervention that should be recommended for couples suffering from any form of infertility. Therefore, counseling should ideally begin before patients start any medical intervention to help with infertility [19]. Some people may require therapy to accept the fact that they are infertile, and the therapist should tell them about all the options available to them. Communication is the focal point in treatment and thus helps better communication among partners. As some studies have indicated, addressing psychological problems such as depression, anxiety and stress may help increase the chance of conception [19]. It has also been known to aid in developing coping mechanisms and decision-making while undergoing treatment. Besides anxiety and depression, patients who suffer from various sleep and eating disorders may also benefit from it. An IVF clinical specialist with expertise in mental health nursing is appropriate for such patients as they know both reproductive procedures and psychotherapy. On the other hand, people with expertise in psychological and social work have a strong foundation in mental health. Still, they lack the medical understanding of reproductive procedures, which is a necessary component for couples receiving infertility treatment. 

Relaxation Techniques

Along with psychological interventions, relaxation techniques have been widely shown to reduce negative emotions in a range of medical patients [20]; more specifically, they have been shown to significantly reduce anxiety scores in women undergoing infertility treatment [21]. Specifically, a study demonstrated that yoga intervention increased the quality of life and decreased negative feelings and thoughts that were associated with infertility [22]. Multiple relaxation techniques recommended by specialists are meditation, deep breathing, guided imagery, and yoga [16]. 

Self-Administered Interventions

Psychological interventions do not necessarily need to be administered by a clinician; there are self-administered options available as well [23]. A randomized controlled prospective study of 166 first-time IVF patients evaluated the use of a self-administered cognitive coping and relaxation intervention (CCRI). The findings suggested that patients utilizing the CCRI displayed more positive reappraisal coping, improved quality of life, and reported less anxiety [24]. Another self-administered tool is the Positive Reappraisal Coping Intervention (PRCI) which helps people take account of the positive aspects of stressful situations [25].

Patient's needs before, during, and after the treatment

Patient's needs before treatment: The infertility team should be aware that as per international estimates, barely one-tenth of patients seeking consultations or planned treatments may actually undergo them [26]. There could be various causes like disinterest of a person, ethical, financial, or personal. Other attributes of the treatment and medical staff that can lead to indifference include disrespect towards patients, being insensitive to different needs of patients, negative staff communication, and making hasty decisions. Before the start of the treatment, the infertility team should address the patient's health beliefs and lifestyle choices. They should encourage them to change their behavior to improve their reproductive health and fertility.

Patient's needs during the treatment: During the treatment, there is a rise in psychological stress, which can be attributed to the fear of treatment failure. Couples whose first IVF cycle doesn't work may discontinue the treatment due to financial burdens, psychological problems, and physical and relational problems between partners. Women experience more distress as compared to men [18]. The most critical phases during this period are retrieval of the ovum, transferring the embryo, and waiting for the pregnancy test to become positive and, thus, associated with increased distress. Educational status, occupational status, psychological support, acceptance, helplessness, and coping are core mediators of infertility stress in men and women [27]. When treatments fail, two in 10 women report depressive symptoms [28,29]. To address the above needs, patients are advised to undergo regular and scheduled counseling, and active participation from both partners is encouraged. 

Patient's needs after the treatment: After treatment, the couple's needs differ for those who have had a successful treatment and those who have not. Needs of those with failed IVF cycles: Data suggest that five years after failed cycles, childless patients are more likely to involve themselves in substance abuse and dependence (of alcohol, tobacco, and benzodiazepines), than those who become parents by adoption or spontaneous conception [30]. Depressive disorders are common after ineffective treatment. Sometimes, it may lead to the ending of marriages. Therefore, couples are encouraged for therapy sessions with a Mental Health Practitioner (MHP). Needs of those with successful treatment: In this group, women experience greater anxiety since they had a difficult conception, have worries related to the viability, gestation, and live birth of the fetus [31]. General recommendations for staff who handle this group of couples consist of sharing information, preparing, discussing, and clarifying worries related to outcomes of their pregnancies [27]. 

Role of infertility team

Patients from all around the world are affected by infertility, which is a life crisis. Patients diagnosed as infertile go through a great deal of emotional upheaval due to their condition, which leads to increased chances of developing anxiety, depression, low self-esteem, and various other mental disorders. It is definitive that infertility leads to significant distress and those psychological interventions are likely to be associated with decreases in depression and increases in pregnancy rates [23]. Relying on patients to self-report themselves is one of the significant difficulties in determining the degree of anguish experienced by infertile couples. Sometimes, couples may fake their emotions. Usually, in the beginning, couples are hopeful and have a positive approach toward the treatment, which is when data collection for evaluating mental stress is done. As the treatment progresses, levels of stress increase. Patients should be encouraged to talk to a therapist regularly, which helps them better deal with such an overwhelming situation. In the past, the prevalence of psychiatric problems among infertile couples was estimated to be 25%-60% [32]. Moreover, the drugs used to treat infertility have psychological side effects. This includes medications like clomiphene citrate and Letrozole, which makes it difficult to distinguish whether psychological symptoms are due to drug or infertility. Infertility may be due to ovarian, tubal, or uterine causes, but sometimes, the cause remains unknown. Not knowing causes frustration in the affected couple, and they may start to obsess over the fact that "something is wrong with their body", which is seen more in women [33]. In some people, lifestyle modifications like increasing physical activity, a healthy diet, and reducing caffeine intake alone may help reverse diagnosis. In contrast, in others, it may act along with IVF treatment. If the IVF treatment fails in the first round, it leads to higher levels of anxiety and depression, which is one reason for the couple to discontinue their treatment. Here, therapy helps the couple by providing them with all the other options, like adoption. Henceforth, psychotherapy plays a significant role in every step from the diagnosis of infertility until after treatment completion, whether successful or not. In our society, taking therapy for good mental health is still considered a stigma, which is why patients feel hesitant about it. Besides this, a majority of highly distressed patients resist entering psychological treatments mainly due to cost factors and personal factors (they feel they can manage their worries or have less information on when and where to seek help) [34,35]. It is also essential for patients to be available for therapy during IVF and intrauterine insemination, and their desire to receive psychological treatment is the first matter that needs to be delicately addressed by the medical staff. The infertility team needs to communicate clearly that an option of psychological counseling and therapy is available to the couple to aid them in coping with infertility and the treatment process rather than exploring hidden personal failures or psychogenic causes of infertility. Contacting distressed couples personally increases take-up rates of therapy [34-37]. Availing therapy would aid the couple in reducing emotional distress and developing new perspectives and scope of action rather than submitting to unrealistic expectations, blame, guilt, resignation, and hopelessness [38,39]. 

## Conclusions

This article reviewed the available literature to understand the complex relationship between infertility and its psychological impact on couples undergoing the treatment. We viewed that being diagnosed as infertile can be an emotional experience for couples and cause various psychological problems like anxiety, depression, eating disorders, low self-confidence, etc. Other issues include social isolation, decreased communication between partners and difficulties in marriage altogether. This is where the role of psychotherapy comes in. It has been well known that interventions like counselling and psychotherapy for infertile couples have the power to decrease psychological problems such as anxiety and depressive disorders and considerably enhance the conception rates. In some couples who did not have a positive outcome in IVF treatment or are not financially well, therapy helps them provide other options for becoming parents, like adoption. In the end, we deduce that the relationship between stress and infertility is a vicious cycle; couples undergoing infertility treatment are under tremendous pressure, and stress is one of the causes for couples not being able to conceive. Therefore, psychotherapy given in the form of counselling is crucial for such couples and should ideally be started when the infertility diagnosis is made to break the cycle of stress. 
